# Prenatal diagnosis and genetic counseling of a paternally inherited chromosome 15q11.2 microdeletion in a Chinese family

**DOI:** 10.1186/s13039-022-00605-1

**Published:** 2022-07-04

**Authors:** Wenjuan Tang, Guowei Chen, Jingshu Xia, Ying Zhang

**Affiliations:** 1 Department of Maternal Health Care, Shiyan Maternal and Child Health Hospital, Shiyan, Hubei People’s Republic of China; 2 College of Life Science and Technology, Huazhong University of Science and Technology, Wuhan, Hubei People’s Republic of China; 3 Law and Business College of Hubei University of Economics, Wuhan, Hubei People’s Republic of China; 4 Reproductive Medicine Center, Renmin Hospital, Hubei University of Medicine, Shiyan, Hubei People’s Republic of China; 5 Prenatal Diagnosis Center, Renmin Hospital, Hubei University of Medicine, Shiyan, Hubei People’s Republic of China; 6 Hubei Clinical Research Center for Reproductive Medicine, Shiyan, Hubei People’s Republic of China; 7 Biomedical Engineering College, Hubei University of Medicine, Shiyan, Hubei People’s Republic of China

**Keywords:** Chromosomal microarray analysis (CMA), Chromosomal microdeletions/microduplications, Prenatal diagnosis

## Abstract

**Background:**

Proximal region of chromosome 15 long arm is rich in duplicons that, define five breakpoints (BP) for 15q rearrangements. 15q11.2 microdeletion has been previously associated with developmental delay, mental retardation, epilepsy, autism, schizophrenia and congenital heart defects. The literature on this microdeletion is extensive and confusing, which is a challenge for genetic counselling.

**Case presentation:**

We have performed prenatal diagnosis and genetic counseling of a paternally inherited 15q11.2 microdeletion. In this family, father with normal phenotype and fetus with abnormal phenotype have the same microdeletion.

**Conclusion:**

Chromosomal microdeletions and microduplications are difficult to detect by conventional cytogenetics, combination of prenatal ultrasound, karyotype analysis, CMA and genetic counseling is helpful for the prenatal diagnosis of chromosomal microdeletions/microduplications.

## Introduction

Proximal region of chromosome 15 long arm is rich in duplicons that, define five breakpoints (BP) for 15q rearrangements. Recurrent microdeletions and duplications in the genomic region 15q11.2 between breakpoints 1 (BP1) and 2 (BP2) are present in 0.5% to 1.0% of the population. This region contains four protein-coding genes: *NIPA1, NIPA2, CYFIP1* and *TUBGCP5*. 15q11.2 microdeletion between BP1 and BP2 has been previously associated with developmental delay, mental retardation, epilepsy, autism, schizophrenia and congenital heart defects. The literature on this microdeletion is extensive and confusing, which is a challenge for genetic counselling [1, 2]. Here we report the prenatal diagnosis and genetic counseling of a paternally inherited chromosome 15q11.2 microdeletion in a Chinese family.

## Case report

**Fig. 1:**
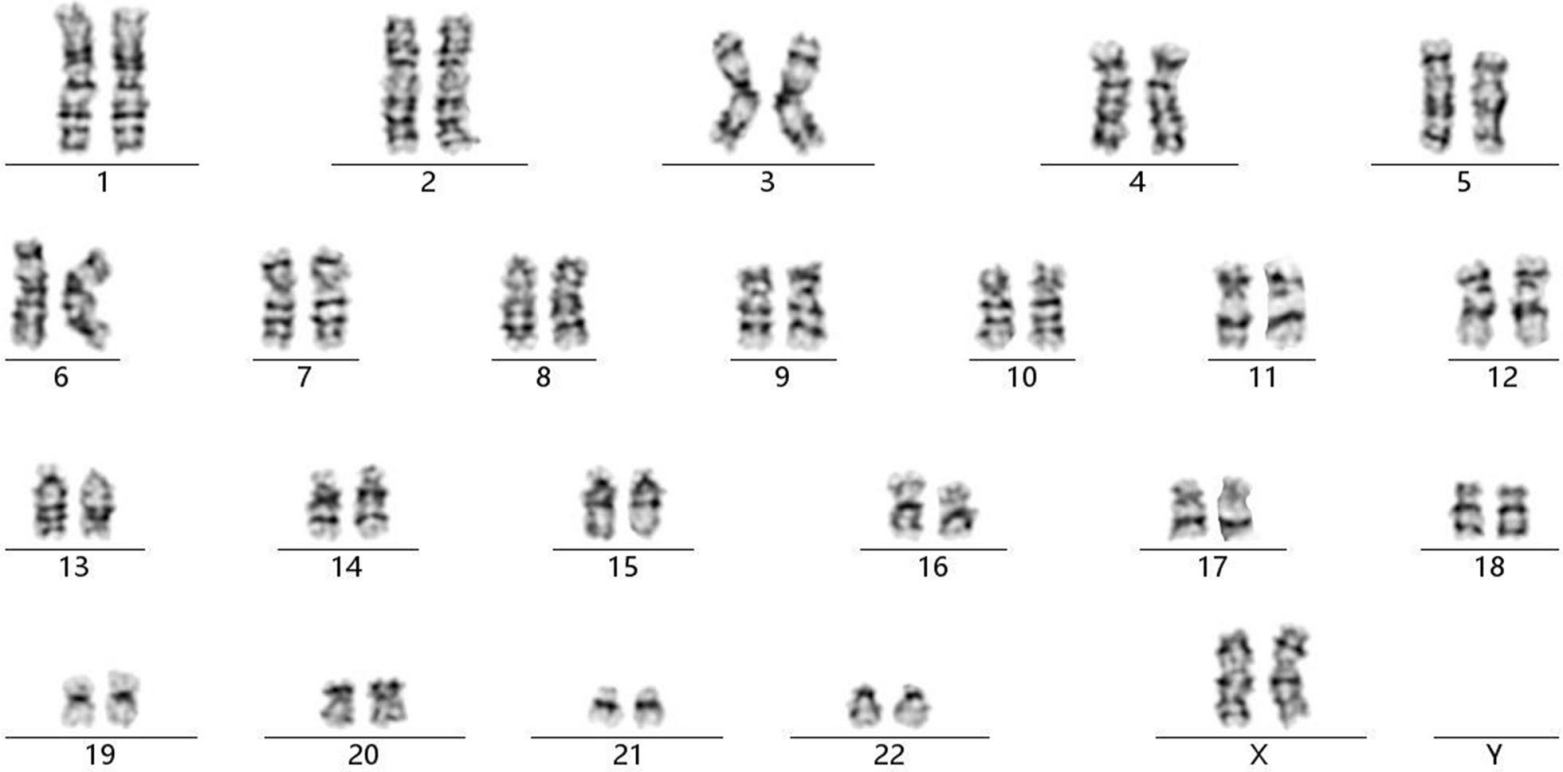
The karyotype of 46,XX

**Fig. 2:**
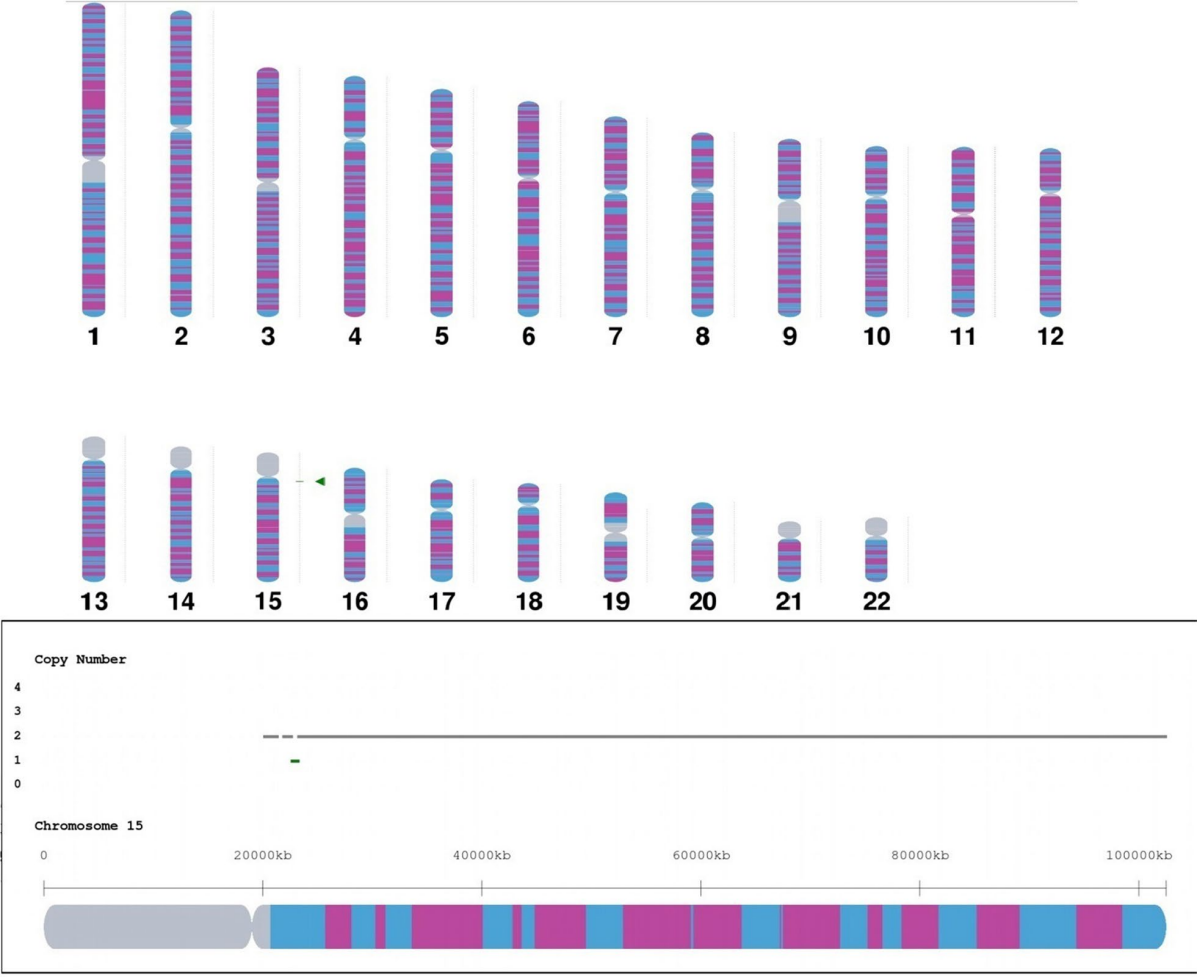
CMA detected a 550-Kb chromosomal microdeletion in the region of 15q11.2(arr[GRCh37]15q11.2(22,676,624_23,226,623)×1)

In 2020, a 35-year-old, gravida 1, para 0, woman underwent amniocentesis because of advanced maternal age at 18 weeks of gestation. There was no family history of birth defects or genetic diseases. Cytogenetic analysis of the cultured amniocytes revealed a normal karyotype of 46,XX (Fig. 1). Chromosomal microarray analysis (CMA) on uncultured amniocytes was performed using the Affymetrix CytoScan 750K chip, which includes 550k non-polymorphic markers and 200k SNP markers. CMA detected a 550-Kb chromosomal microdeletion in the region of 15q11.2, which is to be reported according to International System of Cytogenomic Nomenclature 2020 (ISCN 2020) [3] as arr[GRCh37] 15q11.2(22,676,624_23,226,623) × 1 (Fig. 2). Then we performed both CMA and conventional karyotyping using the samples from the parents' peripheral blood. Their karyotypes were normal. The CMA results showed the father had the same microdeletion as the fetus. SNP markers in the Affymetrix CytoScan 750K chip confirmed a paternal origin of the 15q11.2 microdeletion. We performed a comprehensive physical examination of the parents and failed to identify anything abnormal. After genetic counseling, the parents decided to continue the pregnancy. At 26 weeks of gestation, ultrasound examination showed intrauterine growth restriction (IUGR) and congenital heart defects (pulmonary veins dislocation) in the fetus. After genetic counseling again, the parents decided to terminate the pregnancy, and a female fetus was delivered. The result of autopsy showed the IUGR and congenital heart defects consistent with the prenatal diagnosis.

## Discussion

The 15q11.2 BP1-BP2 microdeletion (Burnside–Butler) syndrome is emerging as a vital pathogenic factor of congenital heart disease [1] and as the most frequent pathogenic copy number variation (CNV) in humans associated with neurodevelopmental disorders with changes in brain morphology, behavior, and cognition [4]. Due to incomplete penetrance and variable expressivity, not all individuals with this microdeletion will present with these typical clinical manifestations [5], and microdeletions or duplications in 15q11.2 are present in 0.5% to 1.0% of the population [2], so more research is needed on causation and genetic counseling.

The 15q11.2 BP1–BP2 microdeletion syndrome has a reported de novo frequency between 5 and 22%, with 51% having inherited the microdeletion from an apparently unaffected parent and 35% having inherited the microdeletion from an affected parent [6].

Most researchers consider that microdeletions or duplications in the region 15q11.2 are pathogenic variations [7, 8]. Some researchers conclude that the pathogenicity of 15q11.2 BP1-BP2 deletions or duplications is low. Their data show that 15q11.2 BP1-BP2 deletions and duplications are common findings among affected and unaffected populations, indicating their low pathogenicity and minimally increased risk for abnormal phenotypes.

Unbalanced chromosome abnormalities (UBCA) are either gains or losses of large genomic regions, but the affected person is not or only minimally clinically affected. John Barber’s research about four families with directly transmitted UBCA indicate that incomplete penetrance and variable expression are features of both sub-microscopic CNVs and UBCAs with relatively low gene and high benign CNV content [9]. The study of these families may help identify further regions that are segmentally dosage insensitive, modifiers of other structural variation or subject to incomplete penetrance and variable expressivity.

Hence, reporting these CNVs and UBCAs in the prenatal setting should be discussed with couples before testing. They suggest that opting out of reporting these CNVs and UBCAs both to clinicians as well as to couples should be considered [9–11].

In this study, the chromosomal deletion is associated with 15q11.2 microdeletion syndrome, the deleted region of 15q11.2 contained a lot of genes, just as *NIPA1, NIPA2, CYFIP1, TUBGCP5* and so on. The *TUBGCP5* gene is associated with the chromosome 15q11.2 deletion syndrome and obsessive–compulsive disorder when disturbed. It also plays a role in microtubule nucleation at the centrosome in cells. The *CYFIP1* gene encodes a protein product that interacts with FMRP, the protein coded by the *FMR1* gene causing fragile X syndrome. The *NIPA1* gene causes autosomal dominant hereditary spastic paraplegia and postural disturbances when disturbed and functions as a magnesium transpor. Mutations of the *NIPA2* gene are reported in patients with childhood absence epilepsy with decreased intracellular magnesium concentration in neurons [4, 12].

In this case, the father carries the same microdeletion and has a normal phenotype, but prenatal ultrasound showed IUGR and congenital heart defects in the fetus. After genetic counseling, the parents decided to terminate the pregnancy.

To summarize, we present a case of paternally inherited microdeletion of chromosome 15q11.2 with IUGR and congenital heart defects. Our case can be helpful for prenatal diagnosis and genetic counseling. Chromosomal microdeletions and microduplications are difficult to detect by conventional cytogenetics. Combination of prenatal ultrasound, karyotype analysis, CMA and genetic counseling is helpful for the prenatal diagnosis of chromosomal microdeletions/microduplications [13].

## Data Availability

Please contact the corresponding author for data requests.
